# Prothrombin Time and Activated Partial Thromboplastin Time Testing: A Comparative Effectiveness Study in a Million-Patient Sample

**DOI:** 10.1371/journal.pone.0133317

**Published:** 2015-08-11

**Authors:** Manu N. Capoor, Jerry L. Stonemetz, John C. Baird, Fahad S. Ahmed, Ahsan Awan, Christof Birkenmaier, Mario A. Inchiosa, Steven K. Magid, Kathryn McGoldrick, Ernesto Molmenti, Sajjad Naqvi, Stephen D. Parker, S. M. Pothula, Aryeh Shander, R. Grant Steen, Michael K. Urban, Judith Wall, Vincent A. Fischetti

**Affiliations:** 1 Department of Bacterial Pathogenesis and Immunology, Rockefeller University, New York, New York, United States of America; 2 Department of Anesthesia, The Johns Hopkins Hospital, Baltimore, Maryland, United States of America; 3 MMF Systems, Inc., New York, New York, United States of America; 4 Department of Anesthesia, Englewood Hospital and Medical Center, Englewood, New Jersey, United States of America; 5 Ludwig-Maximilian University of Munich, Munich, Germany; 6 Westchester Medical Center/New York Medical College, Valhalla, New York, United States of America; 7 Hospital for Special Surgery, New York, New York, United States of America; 8 Department of Surgery, North Shore University Hospital, Manhasset, New York, United States of America; 9 Department of Anesthesia, Washington Hospital Center, Washington, DC, United States of America; 10 Atlantic Health System, Morristown, New Jersey, United States of America; University Hospital Basel, SWITZERLAND

## Abstract

**Background:**

A substantial fraction of all American healthcare expenditures are potentially wasted, and practices that are not evidence-based could contribute to such waste. We sought to characterize whether Prothrombin Time (PT) and activated Partial Thromboplastin Time (aPTT) tests of preoperative patients are used in a way unsupported by evidence and potentially wasteful.

**Methods and Findings:**

We evaluated prospectively-collected patient data from 19 major teaching hospitals and 8 hospital-affiliated surgical centers in 7 states (Delaware, Florida, Maryland, Massachusetts, New Jersey, New York, Pennsylvania) and the District of Columbia. A total of 1,053,472 consecutive patients represented every patient admitted for elective surgery from 2009 to 2012 at all 27 settings. A subset of 682,049 patients (64.7%) had one or both tests done and history and physical (H&P) records available for analysis. Unnecessary tests for bleeding risk were defined as: PT tests done on patients with no history of abnormal bleeding, warfarin therapy, vitamin K-dependent clotting factor deficiency, or liver disease; or aPTT tests done on patients with no history of heparin treatment, hemophilia, lupus anticoagulant antibodies, or von Willebrand disease. We assessed the proportion of patients who received PT or aPTT tests who lacked evidence-based reasons for testing.

**Conclusions:**

This study sought to bring the availability of big data together with applied comparative effectiveness research. Among preoperative patients, 26.2% received PT tests, and 94.3% of tests were unnecessary, given the absence of findings on H&P. Similarly, 23.3% of preoperative patients received aPTT tests, of which 99.9% were unnecessary. Among patients with no H&P findings suggestive of bleeding risk, 6.6% of PT tests and 7.1% of aPTT tests were either a false positive or a true positive (i.e. indicative of a previously-undiagnosed potential bleeding risk). Both PT and aPTT, designed as diagnostic tests, are apparently used as screening tests. Use of unnecessary screening tests raises concerns for the costs of such testing and the consequences of false positive results.

## Introduction

Estimates suggest that 20% to 30% of total American healthcare expenditures may be unnecessary. [1–4] Over-diagnosis of disease has been described as a modern epidemic in high-income countries.[5] A comprehensive review of 146 medical practices found that 40% of those practices recommended when new were reversed upon more rigorous evaluation; some practices were unhelpful, and some were found to substantially increase patient costs without improving outcomes.[6]

Recently, there has been a focus on using objective evidence to combat over-diagnosis and over-treatment of disease.[7] This strategy is motivated by the need to contain medical costs as mandated by the Affordable Care Act; but, also derives from a sense that there are human as well as economic costs to consider when allocating treatment.[8]

Factors that potentially could contribute to higher medical costs include practices that have persisted in medicine and surgery without objective validation of their efficacy. One such practice may be ordering a panel of pre-operative tests that include a prothrombin time (PT) test and/or an activated partial thromboplastin time (aPTT) test prior to surgery to determine whether bleeding is a potential surgical risk.[9–11]

We hypothesize that if PT and aPTT tests are used correctly as diagnostic tests (rather than as screening tests), then there should be specific findings on the patient’s history and physical (H&P) chart to justify such tests. Further, these indications should be consistent with current guidelines as to when PT and aPTT tests should be ordered. Conversely, if PT and aPTT tests are used for screening, then specific findings in a patient’s H&P will not necessarily be present.[12–14] Therefore, we compared each patient’s PT and/or aPTT results with findings on that patient’s H&P to determine whether the tests had been used as diagnostic tests or as screening tests.

## Materials and Methods

We utilized a web-based patient information warehouse and designed a tool for our comparative-effectiveness research. The warehouse is used by hospitals to manage information required for scheduled surgeries that originates with their affiliated surgeons. Our research tool provided us access to de-identified and aggregated patient information through appropriate de-identification provisions (e.g., via hospital service agreements). [15, 16] This research was reviewed and approved by the Rockefeller University Institutional Review Board (MCA-0669) on June 10, 2014.

De-identified pre-surgical patient data (H&Ps and lab reports), generated at 19 hospitals and 8 associated ambulatory surgery centers (Table 1) were aggregated. The time period covered was 48 months (from 2008 through 2012), with the exception of facilities 6, 16, 22, and 27 (data aggregated for 36 months) and facilities 9 and 24 (data aggregated for 46 months).

**Table 1:** H&P and Lab Data of Patients Who Underwent Surgery.

Facility		Facility Location	Facility Type	Patients	H&Ps	%	Labs	%	H&Ps & Labs	%	Data Set	%
**Tertiary HospitalsS**	1	Delaware	Tertiary Hospital	78,473	62,176	79%	47,738	61%	43,483	55%	62,176	79%
	2	New Jersey	Tertiary Hospital	23,846	8,397	35%	19,200	81%	7,325	31%	8,397	35%
	3	New Jersey	Tertiary Hospital	40,439	27,780	69%	20,792	51%	18,579	46%	27,780	69%
	4	Maryland	Tertiary Hospital	62,300	21,639	35%	22,360	36%	13,660	22%	21,639	35%
	5	New York	Tertiary Hospital	46,562	37,201	80%	36,680	79%	33,444	72%	37,201	80%
	6	Florida	Tertiary Hospital	14,554	11,199	77%	11,248	77%	9,726	67%	11,199	77%
	7	New Jersey	Tertiary Hospital	41,733	30,161	72%	20,570	49%	17,163	41%	30,161	72%
	8	New Jersey	Tertiary Hospital	62,234	46,570	75%	38,040	61%	35,028	56%	46,570	75%
	9	New York	Tertiary Hospital	55,670	21,455	39%	15,108	27%	11,326	20%	21,455	39%
	10	New Jersey	Tertiary Hospital	29,355	19,960	68%	19,840	68%	15,752	54%	19,960	68%
	11	Pennsylvania	Tertiary Hospital	28,079	19,023	68%	14,768	53%	13,790	49%	19,023	68%
	12	D. of Columbia	Tertiary Hospital	57,865	35,278	61%	16,877	29%	15,080	26%	35,278	61%
	13	Florida	Tertiary Hospital	25,518	19,590	77%	16,200	63%	15,260	60%	19,590	77%
	14	New York	Tertiary Hospital	31,438	19,596	62%	11,908	38%	11,169	36%	19,596	62%
	15	Delaware	Tertiary Hospital	22,859	17,672	77%	11,230	49%	10,114	44%	17,672	77%
	16	New York	Tertiary Hospital	16,804	12,192	73%	5,129	31%	4,236	25%	12,192	73%
	17	New York	Orthopedic Hospital	113,646	72,806	64%	30,833	27%	24,531	22%	72,806	64%
	18	New York	Eye and Ear Hospital	42,311	33,359	79%	27,955	66%	27,229	64%	33,359	79%
	19	Massachusetts	Eye and Ear Hospital	59,407	53,525	90%	31,094	52%	30,857	52%	53,525	90%
Subtotal				853,079	569,579	67%	417,570	49%	357,752	42%	569,579	67%
**AmbSurg Centers**	20	Delaware	Hospital 1 Surgery Center	37,414	28,732	77%	15,497	41%	14,302	38%	28,732	77%
	21	Maryland	Hospital 4 Surgery Center	26,411	11,385	43%	9,065	34%	5,968	23%	11,385	43%
	22	Florida	Hospital 6 Surgery Center	9,737	2,539	26%	2,549	26%	1,850	19%	2,539	26%
	23	New Jersey	Hospital 8 Surgery Center	27,392	19,148	70%	9,652	35%	8,509	31%	19,148	70%
	24	New York	Hospital 9 Surgery Center	29,469	12,215	41%	7,286	25%	5,902	20%	12,215	41%
	25	New Jersey	Hospital 10 Surgery Center	18,247	10,123	55%	7,719	42%	6,324	35%	10,123	55%
	26	Delaware	Hospital 15 Surgery Center	21,001	16,377	78%	8,870	42%	8,337	40%	16,377	78%
	27	New York	Hospital 16 Surgery Center	30,708	11,951	39%	3,777	12%	3,054	10%	11,951	39%
Subtotal				200,379	112,470	56%	64,415	32%	54,246	27%	112,470	56%
Total				1,053,472	682,049	65%	481,985	46%	411,998	39%	682,049	65%

The research tool accessed scheduling systems for elective surgery at each hospital. In-patient and emergency room patients who underwent surgeries were not included; such patient information resides on in-house hospital systems, not accessible to the tool. This approach yielded patient information on a consecutive sample of 1,053,472 patients, representing every patient admitted for elective surgery between 2009 and 2012 at all 27 settings, provided that these patients were scheduled, confirmed, and actually underwent surgery (Table A in S1 File).

Joint Commission requirements mandate that all hospitals have a recent H&P in place for every patient scheduled for surgery. Therefore, each hospital has an H&P for each patient either in the data warehouse (from its surgeons) or on their own EHR system[17] Lab tests are not required for surgery and may not be available on the research tool.

**Fig 1:**
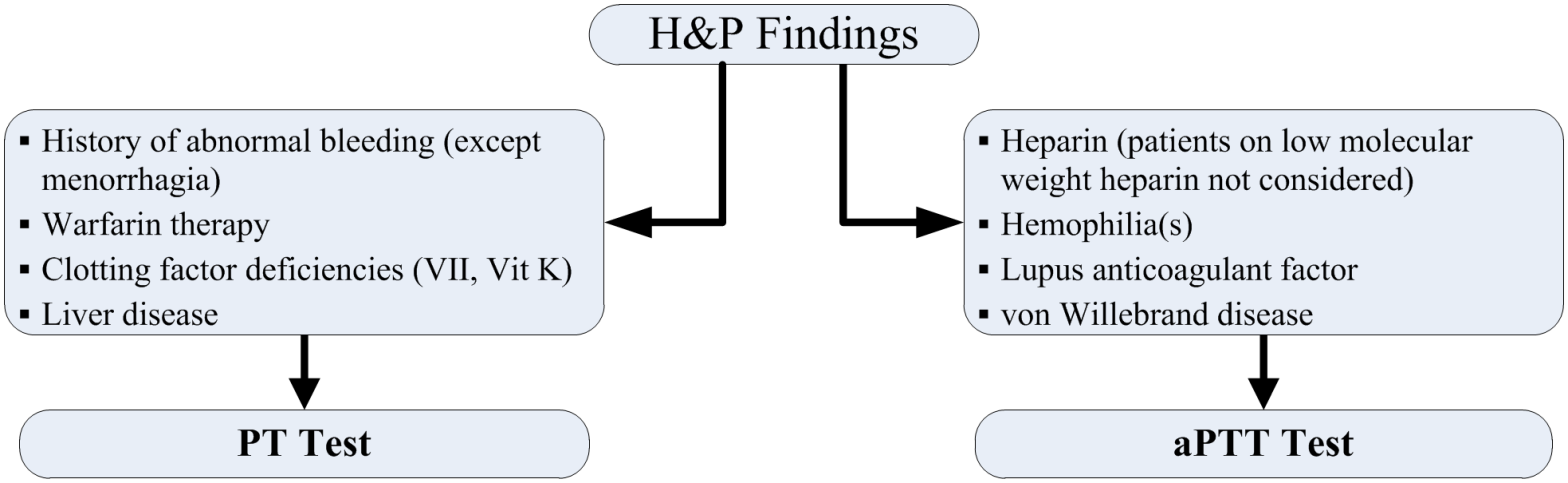
H&P Findings That Prompt PT and aPTT Testing.

Patient H&P records were evaluated to determine whether there was justification for PT and aPTT testing. Unnecessary tests were defined as PT tests done on patients without a history of: 1) abnormal bleeding, 2) warfarin therapy, 3) vitamin K-dependent clotting factor deficiency, or 4) liver disease; or aPTT tests done on patients without a history of: 1) heparin use, 2) hemophilia, 3) antiphospholipid antibodies (lupus anticoagulant), or 4) von Willebrand disease (Fig 1).

## Results

Our research tool included 682,049 H&Ps from the 1,053,472 patient records in surgeon EHRs. Thus 65% of patients had H&Ps in our data set (Table 1). The remaining 371,423 H&Ps were in-hospital EHRs and therefore not available for analysis.

**Fig 2:**
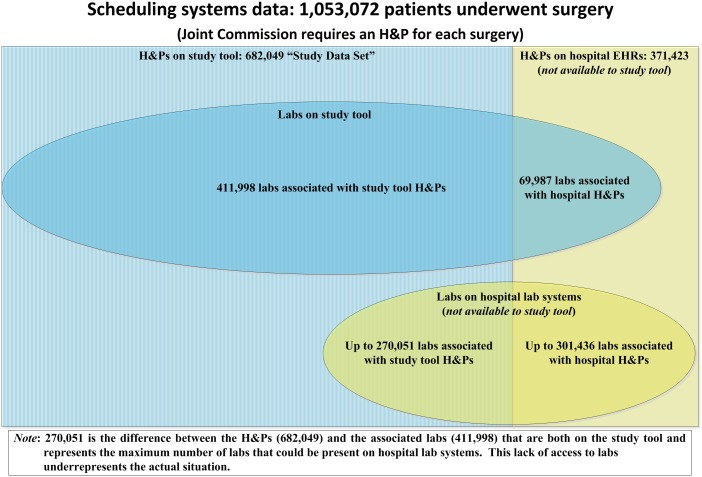
Origin of Study Data.

Among the 682,049 H&Ps, we found 411,998 associated with PT and aPTT tests (60.4%) (Fig 2). Some of the remaining 270,051 surgeries may have had associated labs on hospital lab systems that were not accessible to us; therefore, this analysis under-represents the actual ratio of labs to H&Ps. Roughly 39.1% of all potential records (411,998/1,053,472) were evaluated in this study. We cannot assess how many patients received PT and aPTT testing whose records are not available to us.

**Fig 3:**
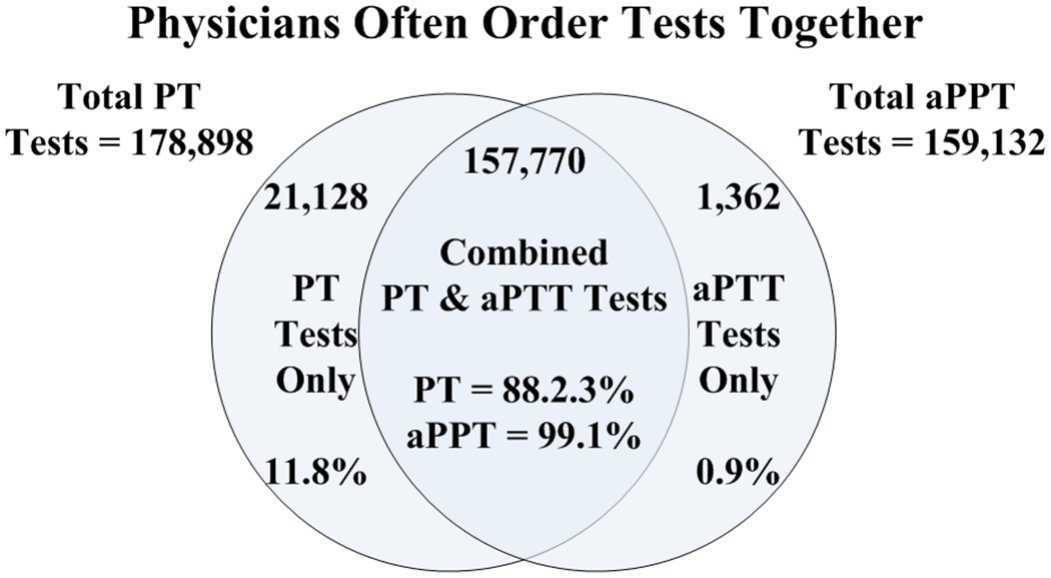
Distribution of PT Only, aPTT Only, and Combined PT/aPTT Tests.

Roughly 26.2% of all pre-surgical patients accessible in the database received PT tests, of which 94.3% of tests were deemed unnecessary, given the absence of findings on the H&P (Table 2); this means that at least 158,378 unnecessary PT tests were done. Similarly, 23.3% of all pre-surgical patients received aPTT tests, of which 99.9% were deemed unnecessary given an absence of H&P findings (Table 2); this is equivalent to at least 149,484 unnecessary aPTT tests. In most cases, PT and aPTT tests were ordered together (Fig 3). The PT test was ordered 178,898 times, and the aPTT test was ordered 159,132 times. The tests were ordered together in 157,770 instances. This represents 88.2% of all PT tests ordered, and 99.1% of all aPTT tests ordered. The aPTT test was ordered on its own in only 1362 cases.

**Table 2:** Unnecessary Testing—Where H&Ps Show No Findings.

Facility		Data Set	PT Tests Ordered and % of Data Set		H&Ps Showing no PT Findings (Unnecessary Tests)		aPTT Tests Ordered and % of Data Set		H&Ps Showing no aPTT Findings (Unnecessary Tests)	
**Tertiary Hospitals**	1	62,176	9,881	15.9%	8,811	89.2%	4,240	6.8%	4,238	100.0%
	2	8,397	4,833	57.6%	4,549	94.1%	4,753	56.6%	4,751	100.0%
	3	27,780	9,913	35.7%	9,255	93.4%	9,173	33.0%	9,170	100.0%
	4	21,639	10,077	46.6%	9,591	95.2%	8,235	38.1%	8,231	100.0%
	5	37,201	29,005	78.0%	28,005	96.6%	27,376	73.6%	27,366	100.0%
	6	11,199	2,707	24.2%	2,459	90.8%	2,391	21.4%	2,388	99.9%
	7	30,161	8,114	26.9%	7,698	94.9%	7,939	26.3%	7,927	99.8%
	8	46,570	11,892	25.5%	10,843	91.2%	10,441	22.4%	10,433	99.9%
	9	21,455	2,283	10.6%	2,020	88.5%	2,117	9.9%	2,109	99.6%
	10	19,960	10,281	51.5%	9,787	95.2%	9,979	50.0%	9,974	99.9%
	11	19,023	3,886	20.4%	3,278	84.4%	3,790	19.9%	3,787	99.9%
	12	35,278	4,983	14.1%	4,701	94.3%	4,426	12.5%	4,423	99.9%
	13	19,590	9,000	45.9%	8,704	96.7%	8,397	42.9%	8,396	100.0%
	14	19,596	7,798	39.8%	7,449	95.5%	7,384	37.7%	7,380	99.9%
	15	17,672	2,096	11.9%	2,008	95.8%	1,235	7.0%	1,234	99.9%
	16	12,192	2,446	20.1%	2,314	94.6%	2,403	19.7%	2,400	99.9%
	17	72,806	12,456	17.1%	12,029	96.6%	11,983	16.5%	11,958	99.8%
	18	33,359	21,285	63.8%	20,729	97.4%	19,770	59.3%	19,768	100.0%
	19	53,525	5,050	9.4%	4,148	82.1%	3,555	6.6%	3,551	99.9%
		569,579	167,986	29.5%	158,378	94.3%	149,587	26.3%	149,484	99.9%
**AmbSurg Centers**	20	28,732	563	2.0%	510	90.6%	383	1.3%	383	100.0%
	21	11,385	3,654	32.1%	3,460	94.7%	2,999	26.3%	2,997	99.9%
	22	2,539	243	9.6%	223	91.8%	208	8.2%	208	100.0%
	23	19,148	1,325	6.9%	1,267	95.6%	1,136	5.9%	1,136	100.0%
	24	12,215	853	7.0%	787	92.3%	792	6.5%	788	99.5%
	25	10,123	2,919	28.8%	2,776	95.1%	2,764	27.3%	2,762	99.9%
	26	16,377	520	3.2%	491	94.4%	458	2.8%	458	100.0%
	27	11,951	835	7.0%	811	97.1%	805	6.7%	805	100.0%
Subtotal		112,470	10,912	9.7%	10,325	94.6%	9,545	8.5%	9,537	99.9%
Total		682,049	178,898	26.2%	168,703	94.3%	159,132	23.3%	159,021	99.9%

There is a wide range between facilities in the frequency with which PT and aPTT tests are ordered. For example, facilities 18 and 19 are both eye and ear specialty hospitals; hospital 18 ordered PT tests for 63.8% of patients, while hospital 19 ordered the same tests for 9.4% of patients.

Across all hospitals and centers, the proportion of unnecessary PT tests ranged from 82.1% to 97.4% and the proportion of unnecessary aPTT tests ranged from 99% to 100% (Table 2). Extrapolating the lowest of these proportions to all patients, some of whom had no H&P records available, enables us to calculate that 90.0% of all patients may have received unnecessary PT tests, and 99.6% of all patients may have received unnecessary aPTT tests (Table 2).

**Fig 4:**
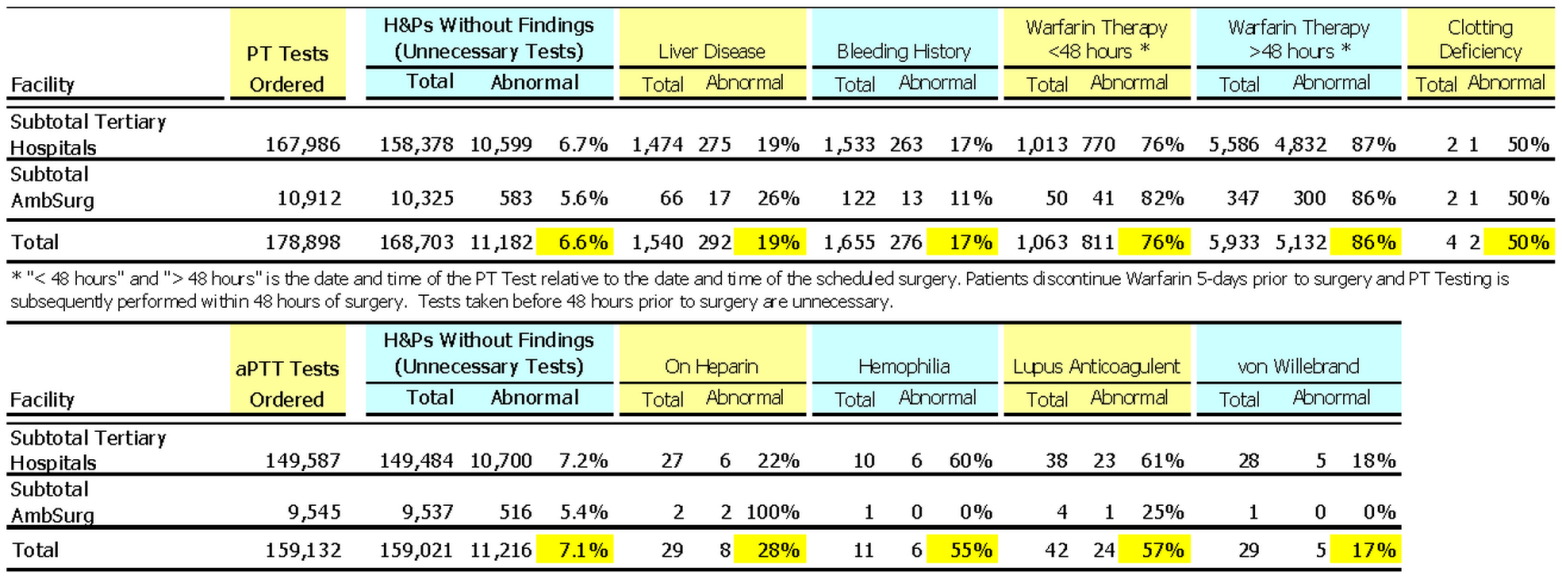
Abnormal Findings for PT and aPTT Tests.

The number and proportion of unnecessary PT and aPTT tests that nevertheless produced abnormal findings is shown (Fig 4). The rate of abnormal test results was significantly higher in patients with relevant findings on their H&P than in patients with no relevant findings. There were a substantial number of patients for whom unnecessary tests were positive (6.6% and 7.1%). We lack sufficient information to tell whether these results are unanticipated true positives or false positives.

The rate of abnormal test results declined with patient age (Table 3). Abnormal PT and aPTT tests were nearly 3-fold more prevalent in patients younger than 30 years than in patients older than 50 years.

**Table 3:** Age Distribution of Abnormal PT and aPTT Test Rates.

	**PT**	**Tests—Age <30**			**Tests—Age 30–50**			**Tests—Age >50**		
**Facility**	**Tests**	**Total**	**Abnormal**		**Total**	**Abnormal**		**Total**	**Abnormal**	
Subtotal Tertiary Hospitals	167,986	13,225	1,267	9.6%	39,426	2,195	5.6%	115,327	13,278	3.7%
Subtotal AmbSurg	10,912	1,149	76	6.6%	2,545	145	5.7%	7,218	734	1.4%
Total	178,898	14,374	1,343	9.3%	41,971	2,340	5.6%	122,545	14,012	3.4%
	**aPTT**	**Tests—Age <30**			**Tests—Age 30–50**			**Tests—Age >50**		
**Facility**	**Tests**	**Total**	**Abnormal**		**Total**	**Abnormal**		**Total**	**Abnormal**	
Subtotal Tertiary Hospitals	149,587	12,611	792	6.3%	36,603	1,832	5.0%	100,369	8,116	2.3%
Subtotal AmbSurg	9,545	1,122	62	5.5%	2,331	87	3.7%	6,092	370	0.7%
Total	159,132	13,733	854	6.2%	38,934	1,919	4.9%	106,461	8,486	2.1%

### Statistical Analysis

**Fig 5:**
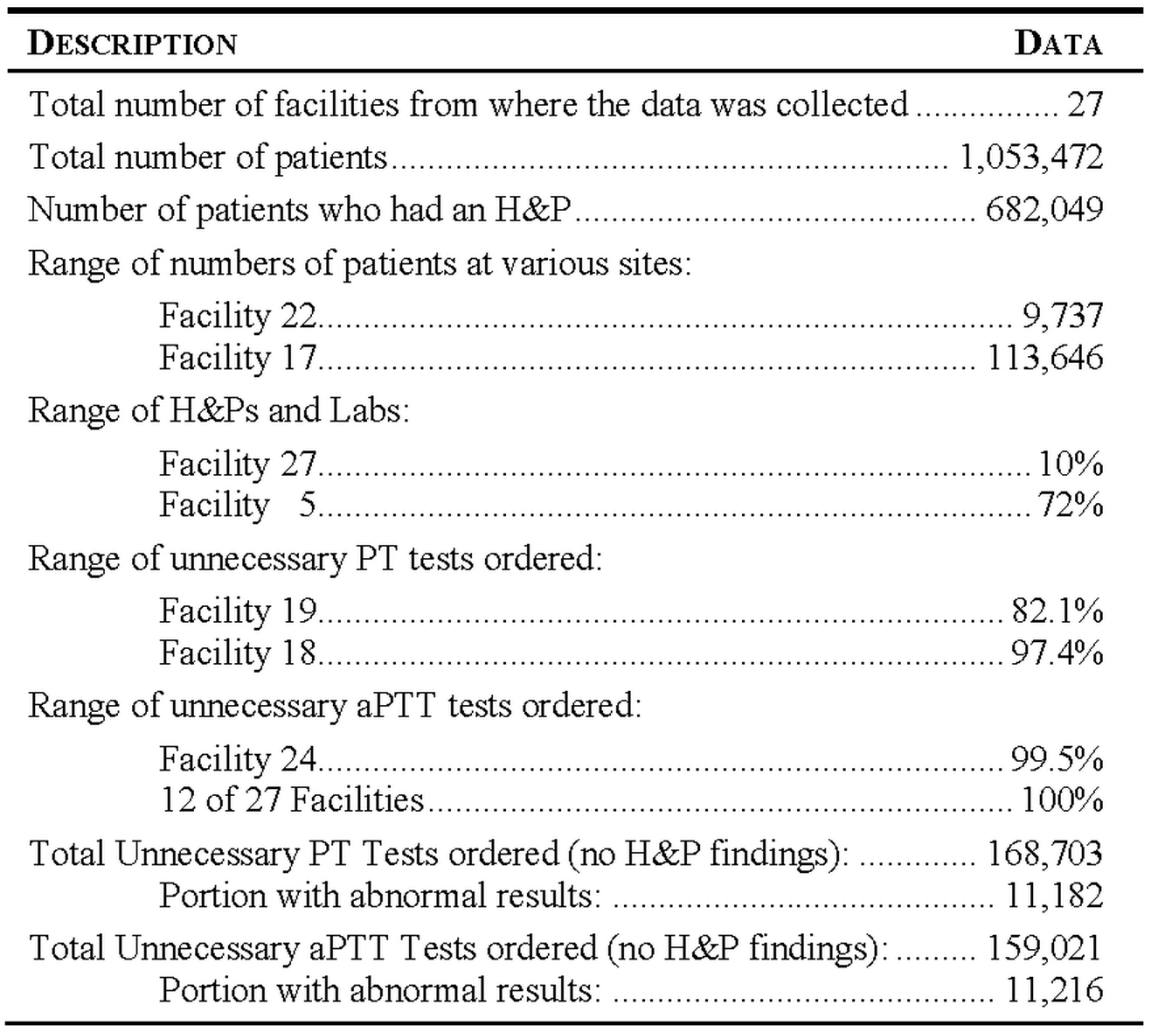
Statistical Overview.

We tested a hypothesis that the percentage of abnormal results on both the PT and aPTT tests is the same (Fig 5). This hypothesis was rejected, the p-value being practically 0. The 95% confidence intervals (PT: 6.48–6.72) and (aPTT: 6.97–7.23) were non-overlapping, indicating that the proportion of false positives on PT and aPTT tests are independent of one another. This suggests that patients who get a false positive on one test are not more likely to get an abnormal result on the other test.

Similarly, a χ^2^ test was used to test the hypothesis that the PT and aPTT tests were used independently. The fourfold contingency table (see Table 4 and Fig 2) for the two tests shows that this hypothesis can be rejected (again the p-value being practically 0), suggesting that physicians tend to order both tests together. This is also evident from the large number of patients who were given both tests or neither test (Table 4).

**Table 4:** Fourfold Contingency Table.

	PT Test Ordered	PT Test Not Ordered	Total
**aPTT Test Ordered**	157,770	1,362	159,132
**aPTT Test Not Ordered**	21,128	501,789	522,917
**Total**	178,898	503,151	682,049

## Discussion

We have demonstrated that 94.3% of all PT tests and 99.9% of all aPTT tests in our data set were ordered without documented justification in patient H&Ps. Our results clearly show that both PT and aPTT tests are routinely used as screening tests (Table 2), although no rationale exists to conclude that these tests are anything other than diagnostic.

Unnecessary PT tests may actually comprise 97.6% rather than 94.3% (Fig 4) if the data is adjusted to eliminate patients on warfarin whose tests were ordered too early (typically patients should be off warfarin therapy five days prior to surgery allowing PT levels to normalize and tested within 24–48 h of surgery to confirm the patient has stopped warfarin therapy). [18]

Though it is well known that these tests are often ordered with no clinical justification, we have shown that this practice of ordering these tests is widespread, at least in the surgical environment.[19] If extrapolated on a national and international level, the scope of unnecessary testing could be significant as well as the direct and indirect healthcare costs and burdens. For instance, the CDC estimates that in the US, there are over 50 million surgical patients operated on annually. [20, 21]

Given the extent to which surgeons order PT and aPTT tests, they must believe that results are important in predicting bleeding complications. The following two questions provide a better perspective regarding these tests.

1. How useful are the tests in predicting bleeding complications?
2. Does an abnormal test result represent a false positives or a true positive?

How useful are the tests in predicting bleeding complications?[22] The PT test was introduced in 1935 for the management of warfarin therapy[23], while the aPTT test was introduced in 1953 and became the test of choice for the management of heparin therapy.[24] Both tests are useful when employed for their intended purposes. However, under most circumstances, even if there is an H&P finding that suggests a need for testing, it is still unlikely that the patient will have a prolonged PT or aPTT as well as a meaningful bleeding complication, since most of the findings on an H&P (disseminated intravascular coagulation, liver disease[25], vitamin K deficiency, congenital factor VII deficiency[26], dysfibrinogenemia[27], factors XII, XI, IX, and VII, lupus anticoagulant, and von Willebrand disease[28–30]) must be quite advanced, and these patients would already have been identified. Accordingly, in contrast with the eight H&P findings commonly used by physicians, the literature only supports the use of these tests where the patient is utilizing warfarin or heparin. Further, near-unanimous results from peer reviewed publications have demonstrated that abnormalities on coagulation tests are not predictive of bleeding events.[31–39]

Does an abnormal test result represent a false positives or a true positive? Abnormal test results in the absence of H&P findings were observed at rates of 6.6% for PT tests and 7.1% for aPTT tests (Fig 4). These findings may be false positives or true positives (i.e. potential bleeding complication in a patient about to undergo surgery). It has been shown that the prevalence of asymptomatic coagulopathies is so low that false-positive test results greatly outnumber true-positive results.[40] Accordingly, an abnormal test result is most likely a false positive for one or more of the following reasons[41]:

- Inadequate determination of reference standards (reagents and instrumentation)[42];
- High patient hematocrit resulting in an artifactual prolongation of the clotting time[43];
- Variations in citrate anticoagulant (3.2% or 3.8%), which is known to affect results[44];
- Fasting state of the patient (plasma turbidity can interfere with optical systems in non-fasting lipemic, hemolyzed, or icteric specimens)[45];
- PT tests should be done within 24 h of collection, and aPTT should be determined within 4 h, especially if the sample is heparinized.[46]

Some weaknesses of the present study may limit the scope of our conclusions. First, our study tool could not access all H&P and lab information from the various sites. Patient information was received from some hospital surgeons who did not utilize the hospital’s in-house EMR and/or lab systems; these patients may constitute a different patient population than other patients in the hospitals studied here. Second, the Joint Commission mandates that an H&P exist for each surgery undertaken. Therefore, the study tool should have had access to an H&P for each of the 1,053,472 surgeries. In contrast, we had access to only 65% of these H&Ps, because all other H&Ps were on hospital systems that were not accessible to our tool. The research tool also had access to only some labs, which underrepresents the actual ratio of H&Ps to labs (Fig 1). Third, on a geographic basis, our sample reflects surgeon ordering practices compiled from data from seven states and the District of Columbia, which may or may not be representative of surgeon ordering practices in the unevaluated portion of the United States. Fourth, the study did not include pregnancy morbidity as an indicator, which is relevant to diagnosing antiphospholipid syndrome (APS) and a potential factor prompting PT and aPTT testing and unexplained thrombosis.

Since a meaningful percentage of surgeons use these tests as screening tests (*88*.*2% of PT tests and 99*.*1% of aPTT tests*) it would appear these tests are being ordered by surgeons as part of their routine pre-surgical process. Accordingly, given the previously mentioned cost burdens, consideration by the relevant industry constituencies could be given to exploring the use of change agents to evolve these apparent pre-surgical processes to conform with evidence-based practices. Some hospitals appear to be proactive in reducing unnecessary testing (*e.g., Table 2: facility 19 vs. facility 18, both specialty eye and ear hospitals*). Our data reflect substantially lower levels of unnecessary testing at some hospitals. For example, one hospital performed roughly 15% as many tests as a comparable hospital with similar surgery cases. The burdens and costs of unnecessary testing may not only refer to the test per se, but also extend to the follow-on professional obligations placed on health-care professionals.

## Conclusions

We report what we believe to be the largest prospective sample of surgical patients ever assembled. Our sample includes 1,053,472 consecutive patients from 27 medical facilities enrolled from 2009 to 2012, and we were able to gather complete data for a subset of 65% of those patients (N = 682,049). Our results show that both PT and aPTT are used as screening tests, though no rationale exists to conclude that these tests are anything other than diagnostic. Overall, 26.2% of patients received PT testing, and 94.3% of those tests were not necessary, given the absence of findings on the patient H&P. Similarly, 23.3% of preoperative patients received aPTT testing, of which 99.9% of tests were unnecessary. For patients with no H&P findings suggestive of bleeding risk, 6.6% of PT tests and 7.1% of aPTT tests were positive, indicating either a false positive or an unanticipated true positive finding. Given that bleeding conditions are likely to be diagnosed symptomatically prior to surgery, most positive findings are likely to be false positives.

We therefore document routine pre-surgical practices for which there is no clinical justification and which can put patients at risk of false-positive findings. Useless tests are clinically inappropriate because they consume resources, yet bring no benefit to patients or clinicians. Empty testing is also ethically wrong, because it puts patients at risk to no purpose. If our study set is representative of national practices in the United States, then modification of current testing practices could substantially reduce the number of unnecessary PT and aPTT tests, thereby saving hospitals, the Centers for Medicare and Medicaid Services, and insurance companies the costs of unnecessary testing. Our tool offers an unprecedented window into unnecessary testing in the United States.

## Supporting Information

S1 File Aggregate Data for H&P and Lab Data. (XLSX)
